# Clinical and genetic characteristics of autosomal recessive polycystic kidney disease in Oman

**DOI:** 10.1186/s12882-020-02013-2

**Published:** 2020-08-14

**Authors:** Intisar Al Alawi, Elisa Molinari, Issa Al Salmi, Fatma Al Rahbi, Adhra Al Mawali, John A. Sayer

**Affiliations:** 1 Translational and Clinical Research Institute, Faculty of Medical Sciences, Newcastle University, Central Parkway, Newcastle upon Tyne, NE1 3BZ UK; 2 National Genetic Center, Ministry of Health, Muscat, Oman; 3 Renal Medicine Department, Ministry of Health, Royal Hospital, Muscat, Oman; 4 Center of Studies and Research, Ministry of Health, Muscat, Oman; 5 Renal Services, Newcastle Upon Tyne Hospitals NHS Foundation Trust, Newcastle upon Tyne, NE7 7DN UK; 6 NIHR Newcastle Biomedical Research Centre, Newcastle upon Tyne, NE4 5PL UK

**Keywords:** Autosomal recessive polycystic kidney disease (ARPKD), Polycystic kidney and hepatic disease 1 (PKHD1), Hepatic fibrosis, molecular diagnosis, Founder alleles

## Abstract

**Background:**

There is a high prevalence of rare genetic disorders in the Middle East, and their study provides unique clinical and genetic insights. Autosomal recessive polycystic kidney disease (ARPKD) is one of the leading causes of kidney and liver-associated morbidity and mortality in Oman. We describe the clinical and genetic profile of cohort of ARPKD patients.

**Methods:**

We studied patients with a clinical diagnosis of ARPKD (*n* = 40) and their relatives (parents (*n* = 24) and unaffected siblings (*n* = 10)) from 32 apparently unrelated families, who were referred to the National Genetic Centre in Oman between January 2015 and December 2018. Genetic analysis of *PKHD1* if not previously known was performed using targeted exon PCR of known disease alleles and Sanger sequencing.

**Results:**

A clinical diagnosis of ARPKD was made prenatally in 8 patients, 21 were diagnosed during infancy (0–1 year), 9 during early childhood (2–8 years) and 2 at later ages (9–13 years). Clinical phenotypes included polycystic kidneys, hypertension, hepatic fibrosis and splenomegaly. Twenty-four patients had documented chronic kidney disease (median age 3 years). Twenty-four out of the 32 families had a family history suggesting an autosomal recessive pattern of inherited kidney disease, and there was known consanguinity in 21 families (66%). A molecular genetic diagnosis with biallelic *PKHD1* mutations was known in 18 patients and newly identified in 20 other patients, totalling 38 patients from 30 different families. Two unrelated patients remained genetically unsolved. The different *PKHD1* missense pathogenic variants were: c.107C > T, p.(Thr36Met); c.406A > G, p.(Thr136Ala); c.4870C > T, p.(Arg1624Trp) and c.9370C > T, p.(His3124Tyr) located in exons 3, 6, 32 and 58, respectively. The c.406A > G, p.(Thr136Ala) missense mutation was detected homozygously in one family and heterozygously with a c.107C > T, p.(Thr36Met) allele in 5 other families. Overall, the most commonly detected pathogenic allele was c.107C > T; (Thr36Met), which was seen in 24 families.

**Conclusions:**

Molecular genetic screening of *PKHD1* in clinically suspected ARPKD cases produced a high diagnostic rate. The limited number of *PKHD1* missense variants identified in ARPKD cases suggests these may be common founder alleles in the Omani population. Cost effective targeted PCR analysis of these specific alleles can be a useful diagnostic tool for future cases of suspected ARPKD in Oman.

## Background

Autosomal recessive polycystic kidney disease (ARPKD) is one of the most frequent cystic kidney diseases in infants and children, causing kidney and liver associated mortality and morbidity. The estimated incidence of ARPKD is 1:6000 to 1:55,000 births [1]. Classically, ARPKD manifests prenatally as enlarged echogenic kidneys with Potter’s syndrome or postnatally during childhood or adolescence with polycystic kidney disease and congenital hepatic fibrosis. It is characterized by bilateral echogenic cystic kidneys caused by dilatation of the renal tubular collecting ducts and congenital hepatic fibrosis secondary to malformation of the liver biliary ducts. ARPKD is also associated with systemic and portal hypertension. Current therapies focus on treating ARPKD symptoms [2].

ARPKD is caused by mutations in the polycystic kidney and hepatic disease 1 (*PKHD1*) (OMIM 606702), which is located on chromosome 6 (p12.3-p12.2). Consistent with the disease phenotype, high expression of *PKHD1* is found in fetal and adult kidney, with low levels detected in the liver, pancreas and arterial walls [3]. The longest open reading frame transcript (NM_138694.4) contains 67 exons and encodes a 4074 amino acid protein called fibrocystin / polyductin. Fibrocystin is an integral membrane protein that is localized to the basal bodies of primary cilia in epithelial cells and hypothesized to control kidney tubular formation by modifying polycystin-2 expression [4]. It was shown that the severity of ARPKD is determined by the type of mutations rather than the location on the *PKHD1* gene [5]. There are over 700 different mutations associated with an ARPKD phenotype [6].

The importance of early diagnosis and management of ARPKD through genetic testing is widely recognized [7]. Precise molecular genetic diagnosis can improve the clinical management of patients, avoiding the exposure to unnecessary and invasive investigations and enhance early detection of kidney and extrarenal complications. The size of *PKHD1* and its heterogeneous mutational spectrum has previously been an obstacle to molecular genetic diagnosis. However, recent advances in massive parallel sequencing / next generation sequencing (NGS) have facilitated routine large scale screening for pathogenic mutations in *PKHD1*.

In addition to mutations in *PKHD1*, mutations in *DZIP1L* (OMIM 671570) may underlie ARPKD. Pathogenic variants have been identified in multiple unrelated consanguineous Turkish, Palestinian and Egyptian patients with ARPKD and a moderate renal phenotype [8]. *DZIP1L* encodes for DAZ-interacting zinc finger protein 1-like protein (DZIP1L) that is located in the centrioles and the distal ends of basal bodies [8].

Inherited kidney disease is a leading cause of end stage kidney disease (ESKD) in children in Oman [7]. In one Omani hospital-based study, the observed birth incidence of ARPKD was evaluated to be much higher than typical, estimated to be 1 in 12,000 births [9]. However, this number was from a selected hospital population and there are no population-based studies evaluating the incidence of ARPKD in the Omani population as a whole. A recent study from Oman showed that hereditary kidney disease accounts for 32% of etiologies causing chronic kidney disease (CKD) in children and ARPKD is the leading cause, accounting for 12% of CKD [10]. The objective of this study was to investigate the clinical features and the molecular genetic diagnosis in patients with suspected ARPKD in an Omani population.

## Methods

### Patients sampling and ethical approval

A group of 40 affected patients, 10 unaffected siblings and 24 parents, from 32 unrelated families were referred to the National Genetic Centre in Oman between January 2015 and December 2018 for the genetic study of ARPKD from the Royal Hospital (*n =* 31), Sohar Hospital (*n =* 8), Rustaq Hospital (*n =* 1) and Al Buraimi Hospital (*n =* 1). Clinical information, including age at clinical diagnosis, use of neonatal ventilation, presence of cystic kidney disease, stage of CKD, splenomegaly, hypertension, urinary concentration defect, urinary tract infection (UTI), pulmonary hypoplasia, oesophageal varices and renal replacement modalities were extracted from the patient’s medical files at the time of genetic testing and were analyzed. Congenital hepatic fibrosis was diagnosed based on clinical parameters, abdominal ultrasonography and where available, pathological results from liver biopsy specimens. The system used for CKD staging is according to that published by the National Kidney Foundation’s Kidney Disease Outcome Quality Initiative KDOQI in 2003 [11].

Additional demographic information including familial history of ARPKD, parental consanguinity and geographical distribution were also obtained. Family pedigrees described in this study were drawn using invitae online tool (https://familyhistory.invitae.com) (Supplementary Figure S1). We have previously reported family structures (but not clinical details) for 5 of these families with genetically proven ARPKD [12].

This study was ethically approved by the Research and Ethical Review and Approval Committee, Ministry of Health in Oman and by the Ethics Committee, College of Medicine and Health Sciences, Sultan Qaboos University in Oman (MREC#1096). All study participants provided written informed consent.

### Mutational analysis

DNA was extracted manually from the peripheral blood lymphocytes of patients and family members using QIAamp DNA mini kit (QIAGEN). For 18 unrelated patients, molecular study had been previously performed through NGS using targeted gene panel that includes cystic kidney disease genes (including *PKD1*, *PKD2*, *PKHD1*, *HNF1B*) (Table S1) [12]. These 18 patients all had biallelic variants in *PKHD1* but mutations were limited to 4 alleles [12]. Based on these previous NGS results, Sanger sequencing screening of the *PKHD1* exons 3, 6, 32 and 58 was performed for another 22 patients from 14 different families with clinically suspected ARPKD. For the confirmation of segregation of variants within families, the available samples of parents, siblings and relatives were also screened using Sanger sequencing (Table 2, Supplementary Figure S1).

For Sanger sequencing, target regions were amplified using AmpliTaq Gold 360 Master Mix kit (Applied Biosystems) and oligonucleotide primers, which were designed using the Primer3 program (http://primer3.ut.ee/). Oligonucleotide primers were designed to cover the whole exon sequence at which variant is located. For large exons, a set of overlapping fragments were amplified using multiple oligonucleotide primer pairs (Supplementary Table S2).

PCR was carried out using the following conditions: denaturation at 95 °C for 15 min; followed by 13 cycles of 94 °C for 30 s, 62 °C for 30 s and 72 °C for 30 s; followed by 8 cycles of 94 °C for 30 s, 46.5 °C for 30 s and 72 °C for 30 s; followed by 16 cycles of 94 °C for 30 s, 54.5 °C for 30 s and 72 °C for 30 s, amplification for a total of 37 cycles, and then elongation at 72 °C for 5 min. PCR products were purified using BigDye Terminator Cycle Sequencing kit (PE Applied Biosystems, Massachusetts, USA) and sequenced (automated ABI 3130).

Sequencing results were aligned and analyzed by comparison with the human reference sequence of the *PKHD1* gene (NM_138694.3) using the SequencePilot 4.2.2 software (JSI Medical Systems GmbH). For genetic analysis all *PKHD1* variants were classified with regard to pathogenicity according to the revised criteria of the American College of Medical Genetics [13].

### Statistical analysis

Statistical analysis was performed using IBM SPSS Statistics 20, with the results expressed as frequencies and percentage for categorical variables and as mean and median for continuous variables, as appropriate.

## Results

### Clinical characteristics

In total, 32 unrelated families were included in this study (Table 1). The cohort includes 40 clinically suspected ARPKD patients (*n =* 19 males and *n =* 21 females), 24 parents and 10 unaffected siblings.

**Table 1:** Clinical characteristics of suspected ARPKD patients from Oman

	N	%
ARPKD patients	40	100
Males	19	48
Females	21	53
***Age at diagnosis:***		
Prenatal	8	20
Birth-1st month	6	15
2–12 months	15	37.5
1–8 years	9	22.5
> 8 years	2	5
***Clinical Features:***		
Systemic hypertension	29	72.5
Congenital hepatic fibrosis	31	77.5
Splenomegaly	19	47.5
Pulmonary hypoplasia	7	17.5
Perinatal deaths (< 28 days)	4	10
Postneonatal deaths (28 days - 1 year)	2	5
Chronic Kidney Disease	24	60
End Stage Kidney Disease	12	30

*ARPKD* Autosomal recessive polycystic kidney disease, *N* Number; %, percentage

Molecular genetic diagnosis of biallelic *PKHD1* mutations was previously known in 18 patients described here and newly identified in a further 20 patients from a total of 30 different families, giving an overall mutation detection rate of 94% in our cohort of clinically suspected ARPKD (Table 2). Twenty-four families (75%) reported a known family history of kidney disease consistent with an autosomal recessive inheritance pattern. Two families remained genetically unsolved, both had no family history of kidney or liver disease. Parental consanguinity was known in 21 families (66%) in keeping with high rates of autosomal recessive disease in Oman.

**Table 2:** Summary of genotype-phenotype outcomes of ARPKD patients with *PKHD1* mutations

Genotype	Pedigree No.	Patient ID	Age at clinical diagnosis	Peri−/ neonatal death	Potter’s phenotype	Pulmonary hypoplasia	Hypertension	CKD	ESRD with RTX	ESRD with CAPD / HD	Hepatic fibrosis	Splenomegaly	Esophageal varices	UTI	Congestive heart failure	Iron deficiency anemia	Molecular diagnostic method
Thr36Met; Thr36Met	1	II1	Prenatal	✔ 4 m	✔		✔ 3 m	✔							✔		NGS*
	2	IV4	2–12 m				✔ 1 y	✔			✔ 1 y	✔					NGS*
	3	II1	1–8 y				✔ 8 y	✔ 8 y		✔	✔ 10 y	✔ 8 y		✔ 8 y			NGS*
	4	II4					✔	✔		✔	✔	✔					NGS*
	5	II1	2–12 m				✔ 2 m	✔ 2 m		✔	✔ 2 m	✔ 2 m	✔				NGS*
	6	II2	Prenatal	✔ 2 m	✔	✔	✔ 1 m	✔ 1 m			✔ 1 m						NGS*
	7	II11	1–8 y				✔ 4 y	✔ 4 y	✔ 13 y		✔ 12 y						NGS*
		III1	1–8 y					✔	✔ 3 y		✔ 3 y						Sanger
	8	II1	1–8 y				✔				✔						NGS*
	9	IV2	Prenatal	✔ < 1 d	✔	✔		✔					✔				NGS*
	10	II1	Birth-1st m				✔ 1 y				✔ 6 y	✔ 6 y		✔ 5 y			Sanger
	11	II1	2–12 m				✔ 2 m	✔ 3 y		✔	✔ 10 m	✔ 2 m					Sanger
		II2	Birth-1st m			✔	✔ Birth	✔ 3 y		✔	✔ 1 y	✔ 1 y		✔ 3 y			Sanger
	12	II1	Birth-1st m				✔ 2 y	✔ 15 d			✔ 5 m	✔ 9 m					Sanger
	13	II6	Birth-1st m	✔ < 1 d	✔		✔	✔							✔		Sanger
	14	II1	Prenatal	✔ 7 d	✔	✔	✔ Birth	✔ 4 d									Sanger
	15	II1	2–12 m					✔ 6 m		✔	✔ 24 y	✔ 24 y					Sanger
	16	II1	Prenatal	✔ < 1 d		✔											Sanger
Thr36Met; Thr136Ala	17	II4	2–12 m				✔ 5 m				✔ 5 m						NGS*
	18	II4	2–12 m				✔ 14 y	✔ 14 y		✔	✔ 7 m	✔ 7 m			✔		NGS*
		II5	Birth-1st m				✔ 11 y				✔	✔			✔		Sanger
	19	II4	Prenatal				✔ 5 m	✔ 2 d			✔ 1 y			✔ 1 y			NGS*
	20	III3	9–13 y				✔	✔ 13 y		✔							NGS*
	21	II1	Prenatal			✔	✔ Birth	✔ 7 y			✔ 18 m	✔ 6 y		✔			Sanger
Thr36Met; Arg1624Trp	22	II2	1–8 y				✔ 12 y				✔ 3 y	✔	✔				NGS*
	23	III4	9–13 y					✔ 12 y	✔ 12 y							✔	NGS*
		III5	2–12 m				✔ 2 y				✔	✔ 5 m					Sanger
		IV2	2–12 m					✔ 10 y	✔ 10 y; 19 y		✔	✔		✔ 10 y		✔	Sanger
	24	II1	2–12 m			✔					✔ 2 y						NGS*
Thr136Ala; Thr136Ala	25	IV1	Birth-1st m				✔ 3 m				✔ 1 y						NGS*
		IV3	Prenatal				✔ 2 m				✔ 1 y						Sanger
Arg1624Trp; Arg1624Trp	26	II2	Birth-1st m				✔ 3 y				✔ 4 y						Sanger
	27	II1	2–12 m				✔ 5 m	✔ 2 y			✔ 5 m	✔ 5 m					Sanger
		II2	2–12 m				✔ 9 m										Sanger
	28	II1	9–13 y								✔	✔					Sanger
		II2	2–12 m				✔ 19 m	✔ 3 y			✔ 3 y						Sanger
	29	II1	1–8 y								✔						Sanger
Arg1624Trp; His3124Tyr	30	II4	1–8 y				✔	✔ 2 y			✔	✔ 25 y	✔			✔	NGS*
Unsolved	31	III3	2–12 m														Sanger
	32	II1	2–12 m								✔	✔					Sanger

Patient ID refers to the identification of patients within pedigree diagram, shown in Supplementary Figure S1. Patients either had a prior molecular genetic diagnosis (denoted NGS) or were diagnosed by targeted exon PCR and Sanger sequencing (denoted Sanger)

*CAPD* continuous ambulatory peritoneal dialysis, *CKD* Chronic kidney disease, *d* Day, *ESKD* End stage kidney disease, *F* Females, *HD* Hemodialysis, *M* Males, *m* Month, *NGS* Next generation sequencing, *RTX* Renal transplantation, *Sanger* Sanger sequencing, *UTI* Urinary tract infections, *y* years

The initial clinical diagnosis was made prenatally in eight patients (20%), during infancy (0–1 year) in 21 patients (52.5%), during early childhood (1–8 years) in 9 patients (22.5%), and in later years (9–13 years) in 2 patients (5%) (Table 1). At the time of molecular diagnosis, clinical features were documented, including systemic hypertension (72.5%), suspected congenital hepatic fibrosis (CHF) (77.5%), CKD (60%) with a median age of onset 3 years, splenomegaly (47.5%), pulmonary hypoplasia (17.5%) and perinatal / post-neonatal death due to respiratory failure (15%) (Table 1). Twelve of patients (27.5%) developed ESKD at median age of 13 years.

### Genetics of Omani ARPKD patients

Our previous NGS study of inherited cystic kidney diseases had identified 4 *PKHD1* missense pathogenic mutations [12] in 18 patients with suspected ARPKD. These included c.107C > T, p.(Thr36Met); c.406A > G, p.(Thr136Ala); c.4870C > T, p.(Arg1624Trp) and c.9370C > T, p.(His3124Tyr) located in exons 3, 6, 32 and 58, respectively. These alleles were found either homozygously or heterozygously in 20 other patients with clinically suspected ARPKD (Table 2). In total, twenty-one families (66%) carried homozygous alleles, while 9 (28%) carried compound heterozygous alleles. The most commonly detected mutation in *PKHD1* was p.(Thr36Met), where 16 families carried it in homozygous state, while 8 families were compound heterozygous for this allele combined with the p.(Thr136Ala) allele (5 families) or the p.(Arg1624Trp) allele (3 families). Four families carried the homozygous mutation p.(Arg1624Trp), whilst one family carried the homozygous mutation p.(Thr136Ala). One family had compound heterozygous mutations p.(Arg1624Trp) and p.(His3124Tyr). The clinical presentations and genotypes are summarized in Table 2. Sanger sequencing chromatograms of the four different mutations in the *PKHD1* gene are shown in Supplementary Figure S2A. The p. (Thr136Ala) has not been reported outside Oman and is absent from reference databases (Table 3).

**Table 3:** Allele frequencies and worldwide distribution of the four *PKHD1* mutations detected in Omani ARPKD patients

Location		Exon 3	Exon 6	Exon 32	Exon 58
Nucleotide variation		c.107C > T	c.406A > G	c.4870C > T	c.9370C > T
Amino acid variation		p.T36M	p.T136A	p.R1624W	p.H3124Y
dbSNP ID		rs137852944	NA	rs200391019	rs1554218666
MAF	ExAC	0.0005193 (63/121312)	not found	0.0001812 (22/121394)	not found
	1000 Genomes Project	0.000199 (1/5008)	not found	not found	not found
	gnomAD (total)	0.0005094 (144/282706)	not found	0.0001379 (39/282816)	not found
	ESP (Exome Variant Server)	0.00031 (4/13006)	not found	0.00015 (2/13006)	not found
	ClinVar (global MAF)	0.0002	not found	NA	NA
	UK 10 K	0.00054 (4/7428)	not found	0.00013 (1/7428)	not found
Genomics England, 100,000G project		16×	not found	6×	not found
*PKHD1* mutation database		86×	not found	15×	4×
Origin		Germany, Caucasian-American, UK, Spain, Czech Republic, Finland, Netherlands, Australia, Oman	Oman	Saudi-Arabia, Caucasian-American, Israel, Netherlands, Czech Republic, Finland-Greece, Oman	Italy, Turkey, Oman
References		Ward et al. (2002) [14] Rossetti et al. (2003) [15] Bergmann et al. (2004) [16] Sharp et al. (2005) [17] Losekoot et al. (2005) [18] Gunay-Aygun et al. (2009) [19] Al Alawi et al. (2019) [12]	Al Alawi et al. (2019) [12]	Onuchic et al. (2002) [20] Gunay-Aygun et al. (2009) [19] Al Alawi et al. (2019) [12]	Furu et al. (2003) [21] Bergmann et al. (2004) [16] Bergmann et al. (2005) [22] Al Alawi et al. (2019) [12]

*dbSNP* Single-nucleotide polymorphism database, *ExAC* Exome Aggregation Consortium, *ESP* NHLBI Exome Sequencing Project (Exome Variant Server), *gnomAD* The Genome Aggregation Database, *MAF* Minor allele frequency, *NA* Not available, 100,000G project, The 100,000 genomes project

### Geographic distribution of *PKHD1* mutations in Oman

**Fig. 1:**
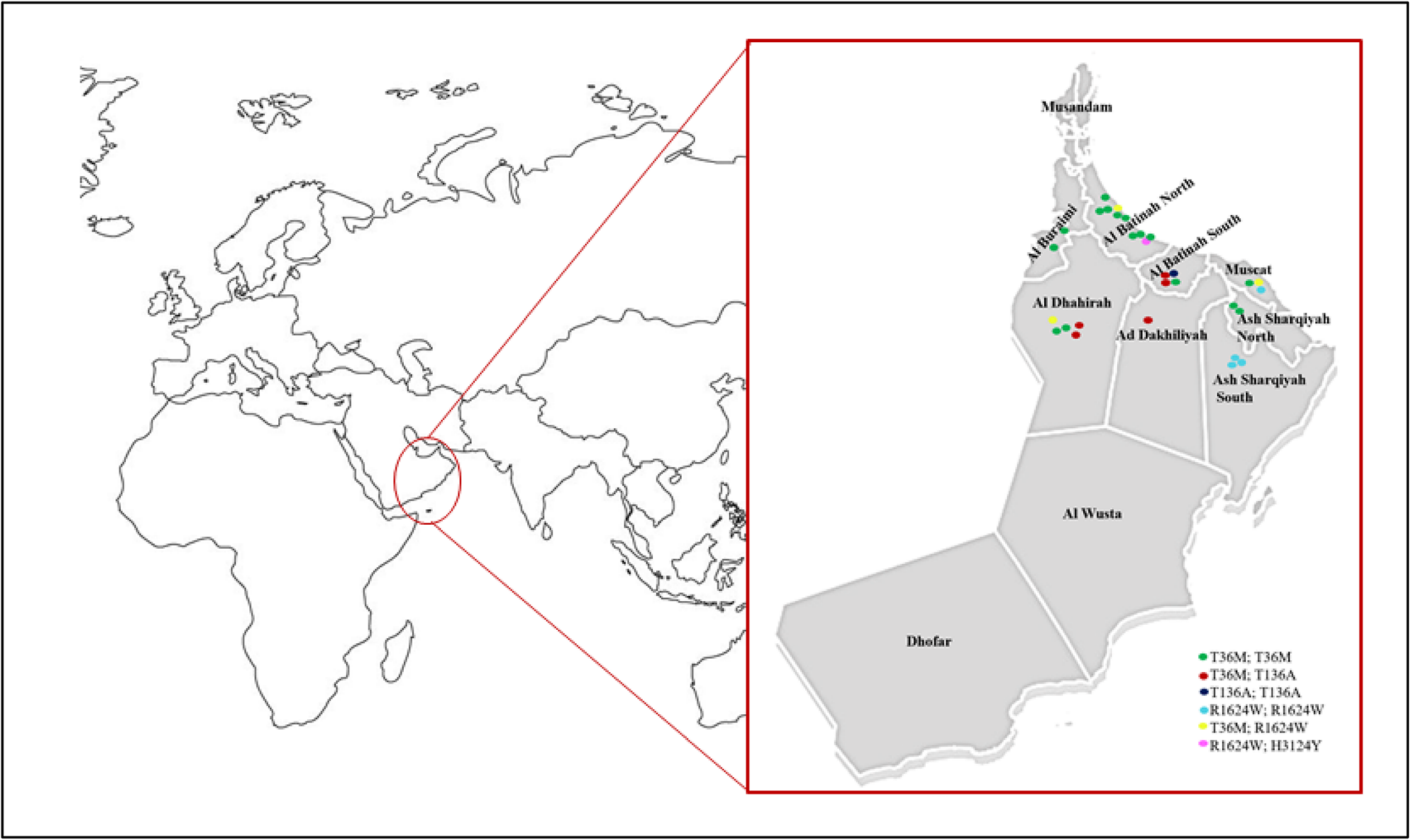
Geographical distribution of the 4 missense *PKHD1* mutations in ARPKD patients from Oman. Each family is presented by plot. Each different mutation is indicated by a different colour. Map sourced through www.freeusandworldmaps.com and modified using www.vectorstock.com software.

The p.(Thr36Met) allele was detected in patients from all governorates of the country except, Musandam, Al Wusta and Dhofar (Fig. 1). The missense p.(Thr136Ala) allele was detected in its homozygous state in one family from Al Batinah South and in heterozygous state in families from Ad Dakhiliyah (*n =* 1), Al Dhahirah (*n =* 2) and Al Batinah south (*n =* 2) (Fig. 1). We postulate that this mutation may be a founder allele in this population.

## Discussion

Inherited kidney diseases, including ARPKD are leading causes of CKD and ESKD in children in Oman, leading to significant morbidity and mortality. Previous studies from Oman have provided ARPKD-associated morbidity data but lacked molecular genetic data [10, 23]. In this study we have provided a clinical and molecular genetic analysis of *PKHD1* in a cohort of 40 patients, obtaining a molecular genetic diagnosis in 38 patients with clinically suspected ARPKD.

Most study patients had early onset ARPKD disease reflected by age at initial diagnosis. Five were diagnosed prenatally, 24 before their first year of life and 11 during childhood. These early-onset phenotypes are in agreement with that reported from other studies [22]. Clinical analysis of our ARPKD patients showed that the frequently associated morbidities were also common in our patients including systemic hypertension, congenital hepatic fibrosis, splenomegaly, pulmonary hypoplasia and CKD. 15% of studied patients died during the perinatal or neonatal period due to respiratory deficiency, which is similar to the death rate during the first year of life reported by Bergmann [22]. It is estimated that 30–50% of ARPKD patients die shortly after birth due to respiratory failure, whereas kidney failure is a rare cause of neonatal death [24]. With the advancement in renal replacement therapy modalities, the survival rate of neonates and children with ARPKD is improved. In our patients, 12 (30%) developed ESKD by median age of 13 years and hence required either renal replacement therapy (*n =* 8) or kidney transplantation (*n =* 4) (Table 2).

As the *PKHD1* is a large gene and in order to identify the causative mutations in our ARPKD patients, we had previously applied targeted NGS gene panel for 18 unrelated patients (Table 2) [12]. Four missense mutations in *PKHD1* were identified as genetic causes of ARPKD in this cohort, with the mutations identified within exons 3, 6, 32 and 58. Therefore, we proceeded with a targeted exon PCR diagnostics approach with Sanger sequencing of these exons alone for the molecular diagnosis of other ARPKD patients (*n =* 20) from 14 different families. In total, 30 out of 32 suspected ARPKD families were solved with biallelic changes in *PKHD1*, achieving a diagnostic rate of 94%, hence providing cost effective targeted PCR analysis of these specific alleles as a convenient diagnostic tool. Failure to detect mutations in two unrelated probands using the Sanger screening approach alone may be explained by the heterogeneity of the ARPKD disease where mutations may lay in other exons or indeed in other recessive cystogenes that phenocopy ARPKD. None of the unsolved patients had have family history of kidney disease, therefore mutations in autosomal recessive genes that lead to ARPKD-like phenotypes such as the *DZIP1L* [8] or even in dominant cystic kidney disease genes such as *HNF1B*, *PKD1* and *PKD2* that often occur de novo are possible [25]. Whole exome sequencing approaches that would allow inclusion of genes such as *DZIP1L* are the suggested option for these unsolved patients. With the current improvements of high throughput sequencing of different renal ciliopathy genes, it is anticipated that the majority of patients with cystic kidney disease phenotypes can receive a precise molecular genetic diagnosis.

To date, 748 unique *PKHD1* variants have been recorded in the Human *ARPKD/PKHD1* Mutation Database (http://www.humgen.rwth-aachen.de/index.php). Approximately 45% of these variants are missense alterations resulting in substitution of conserved amino acids, which usually leads to partial or complete dysfunction of fibrocystin. All of the *PKHD1* variants in this study are missense alterations of highly conserved amino acids. Although three of these variants are reported in the *ARPKD / PKHD1* Mutation Database, p. (Thr36Met) is the most persistent mutation found in *PKHD1* in ARPKD patients to date. Structurally, fibrocystin is an integral membrane protein consisting of a large amino terminal extracellular domain (about 3860 aa) containing various glycosylation sites, a single transmembrane segment and a short cytoplasmic C-terminal tail (about 195 aa) comprising four potential protein kinase A phosphorylation sites [3] (Supplementary Figure S2B). The localization of fibrocystin to primary cilia and its integral structure predicted a sensory role at which fibrocystin acts as receptor transducing the extracellular information into the cell through stimulation of signal cascades, thus controlling cell-cell adhesion and proliferation [3]. The 4 missense variants identified in this study are located in exons encoding the extracellular domain (Supplementary Figure S2C) and are either very rare or not observed in the reference databases of healthy controls (including ExAC, gnomAD, 1000G project (Table 3)).

In the Omani population our previous NGS studies did not find *PKHD1* truncating mutations that have been described in patients with perinatal lethal phenotypes [22, 26]. The most frequent change identified in Omani families was p.(Thr36Met) located in exon 3, detected homozygously in 16 families and heterozygously in 8 families, accounting for almost 75% of the families. Patients with homozygous p.(Thr36Met) change (*n =* 18) had an earlier age of onset and an increased severity of disease (6 had a severe perinatal presentation and died before 1 year of life, while the remaining had either infantile or early childhood presentation leading to ESKD) (Table 2). Consistent with observations made by Bergman et al. [26], the p.(Thr36Met) variant may lead to variability in the age of onset and severity of disease. Additionally, p.(Thr36Met) in combination with the missense changes p.(Thr136Ala) and p.(Arg1624Trp) in some of our cases caused a relatively severe form of ARPKD, which is in agreement with previous reported studies [21, 27, 28].

The relatively common pathogenic *PKHD1* allele p.(Thr36Met) has been described in many populations and ethnicities. Whether this allele is a highly conserved ancestral change that is frequent in some populations such as the Central European population [15, 27], or caused by recurrent mutational events is uncertain [22]. The p.(Thr36Met) allele appears to be common in European genomes, with an expected carrier frequency of 1:412 [29]. However, detections of this change in patients from different ethnicities and origins are suggestive that p.(Thr36Met) is a *PKHD1* ‘hotspot’ mutation caused by the frequent methylation events of cytosine to thymine in the CpG sites [17, 27]. It is also assumed that the substitution of the amino acid Threonine to Methionine creates a potential alternative translation start codon that may be even stronger than the original start codon [21]. The protein product initiating from position c.107 would be predicted to lead to complete loss of protein function due to improper protein folding [21].

The missense change, p.(Arg1624Trp), was previously reported in patients from different ethnicities, including Caucasian Americans [17, 19], Dutch [18], Czech Republicans [28], Slovenians [30], Saudi Arabians [17, 31, 32] and Kuwaitis [33]. The p.(Arg1624Trp) mutation has been described with late onset or older ARPKD presentations when present homozygously [17, 31] and heterozygously in *trans* with other truncating or missense change [17, 30]. In contrast, 6 of our patients with the p.(Arg1624Trp) mutation developed clinical features of ARPKD in infancy, 5 presented during childhood period and 1 at 11 years of age. The p.(His3124Tyr) combined with p.(Arg1624Trp) was found in a 26 year old patient with stage 4 CKD, who was initially diagnosed with polycystic kidney disease in early childhood (Table 2). These findings are in contrast to those made by Bergmann et al. [16] and Gunay-Aygun [19] that correlated p.(His3124Tyr) with a severe perinatal-fatal phenotype.

These results therefore demonstrate that establishing genotype-phenotype correlations in ARPKD is challenging. Any correlation is complicated by the large number of missense variants distributed over the entire length of the coding exons of *PKDH1* and its complex splicing pattern [3]. It was believed that two truncating mutations are associated with severe perinatal lethality and at least the presence of one missense is required for survival beyond the neonatal period. However, evidence is accumulating on the increased pathogenicity of some missense mutations that may cause complete loss of function effects [22]. The wide variability in ARPKD severity among patients may in part be explained by differences in *PKHD1* mutations, influences of modifiers genes and environmental factors [25].

ARPKD is generally a severe form of pediatric ciliopathy with recognized phenotypic variability. While a significant number of ARPKD patients surviving the neonatal period reaches adulthood, some patients have an adulthood presentation and their kidney function ranges from normal to moderate kidney insufficiency to ESKD [34]. Although bilateral kidney enlargement with multiple cysts is the major clinical characteristic, liver manifestations may lead to symptomatic disease complications in ARPKD patients. Liver disease tends to manifest later than kidney disease typically with progressive hepatic fibrosis and portal hypertension [34]. Hypersplenism, portal hypertension, and variceal bleeding are major liver involvements that may develop as a result of progressive liver fibrosis. In rare cases, both kidney and liver disease may present in late adolescence or in adulthood [35]. The low prevalence, limited clinical information and atypical sonographic pattern of adult ARPKD patients can challenge the clinical diagnosis and management, hence genetic testing may be demanded for the establishment of definite diagnosis [36]. In this study, the absence of late presenting ARPKD in our cohort may be due to recruiting predominantly cystic kidney disease phenotypes.

The Omani population is characterized by a unique structure of tribal communities occupying definite geographical regions. This structure is conserved over many generations and has created genetic isolates [37]. The custom of consanguineous marriages as well as within-tribe (endogamous) marriages are extremely conserved in Oman, accounting for 56.3% [38] and 20.4% of total marriages, respectively [39]. Over 300 genetic diseases have been identified in the Omani population [40]. The high frequency of recessive disorders in this population is probably related to a combination of genetic drift, consanguinity, and geographical isolation. The detection of only 4 pathogenic variants in different geographical regions of the country may be explained by the presence of *PKHD1* founder alleles and reveals a high degree of homogeneity in this population. Similarly, genetic studies of population isolates such as Finnish, French, Ashkenazi Jews and Africans represent a powerful method of finding founder mutations in *PKHD1*, which can be utilized for efficient diagnostic testing of at-risk individuals and pregnancies in these populations (Supplementary Table S3) [27, 41–43].

Currently there is no clinical cure for ARPKD other than managing the clinical complications [34]. Together translational research and clinical trials in patients may facilitate successful drug development in coming future. With the absence of clinical biomarkers and lack of comprehensive assessment of the available therapeutic options for ARPKD patients on one hand and great morbidity and mortality of disease on the other hand, there is a serious need for prospective and retrospective population studies and construction of an international clinical database. Such effort can elaborate the current understanding of ARPKD and deliver more information on extrarenal manifestations and treatment options. Recently, the German Society for Pediatric Nephrology (GPN) and the European Study Consortium for Chronic Kidney Disorders Affecting Pediatric Patients (ESCAPE) collaborated to initiate an international multicenter registry of ARPKD (ARegPKD) [44]. The continued identification of *PKHD1* variants and their associated phenotypes is to be promoted and inclusion of cohorts from different ethnicities is valuable and should be encouraged.

## Conclusions

In conclusion, this study shows that prior NGS identification of *PKHD1* mutations and subsequent screening of only 4 exons of the *PKHD1* gene was sufficient to identify the expected causative alleles in 94% of the studied families and was suggestive of founder effects in this gene. There are many advantages for identifying high frequency limited disease associated mutations in a population, including simplifying the diagnostic testing, providing genetic counseling for individuals at risk and allowing rapid detection of mutations in other family members.

## Supplementary information

**Additional file 1 **: **Figure S1**. Pedigrees of the 32 analysed families. **Figure S2**. Representation of the missense variants of the PKHD1 gene detected in ARPKD patients in relation to the gene exon structure and protein domains. **Table S1**. Disease categories and genes selected for targeted NGS panel for cystic kidney disease. **Table S2**. Primers used for PCR amplification and sequencing of PKHD1 gene. **Table S3**. Different PKHD1 founder mutations associated with different ethnicities.

## Data Availability

All data generated or analyzed during this study are included in this published article [and its supplementary information file]. The raw data are available from the corresponding author on reasonable request.

## Abbreviations

ARPKD: Autosomal recessive polycystic kidney disease
CHF: Congenital hepatic fibrosis
CKD: Chronic kidney disease
*DZIP1L*: DAZ-interacting zinc finger protein 1-like
ExAc: Exome Aggregation Consortium
ESCAPE: European Study Consortium for Chronic Kidney Disorders Affecting Pediatric Patients
ESKD: End stage kidney disease
gnomAD: Genome Aggregation Database
GPN: German Society for Pediatric Nephrology
KDOQI: Kidney Disease Outcome Quality Initiative
NGS: Next generation sequencing
OMIM: Online Mendelian Inheritance in Man
*PKHD1*: *P*olycystic kidney and hepatic disease 1
*PolyPhen-2*: Polymorphism Phenotyping v2
SIFT: Sorting Intolerant From Tolerant
UTI: Urinary tract infections
