# Supplementary material for: Clinical and genetic characteristics of autosomal recessive polycystic kidney disease in Oman
Source: BMC Nephrol. 2020 Aug 14;21:347. doi: 10.1186/s12882-020-02013-2 (PMC7429752; doi:10.1186/s12882-020-02013-2)
Supplement: Supplementary file 1 — Additional file 1 : Figure S1. Pedigrees of the 32 analysed families. Figure S2. Representation of the missense variants of the PKHD1 gene detected in ARPKD patients in relation to the gene exon structure and protein domains. Table S1. Disease categories and genes selected for targeted NGS panel for cystic kidney disease. Table S2. Primers used for PCR amplification and sequencing of PKHD1 gene. Table S3. Different PKHD1 founder mutations associated with different ethnicities. [file 12882_2020_2013_MOESM1_ESM.zip › ARPKD ModifiedSupplementary FiguresR2.docx]

**Clinical and Genetic Characteristics of Autosomal Recessive Polycystic Kidney Disease in Oman**

Intisar Al Alawi^1,2^, Elisa Molinari^1^, Issa Al Salmi^3^, Fatma Al Rahbi^3^, Adhra Al Mawali^4^, and John A. Sayer^1,5,6^

**Supplementary Figures**

**
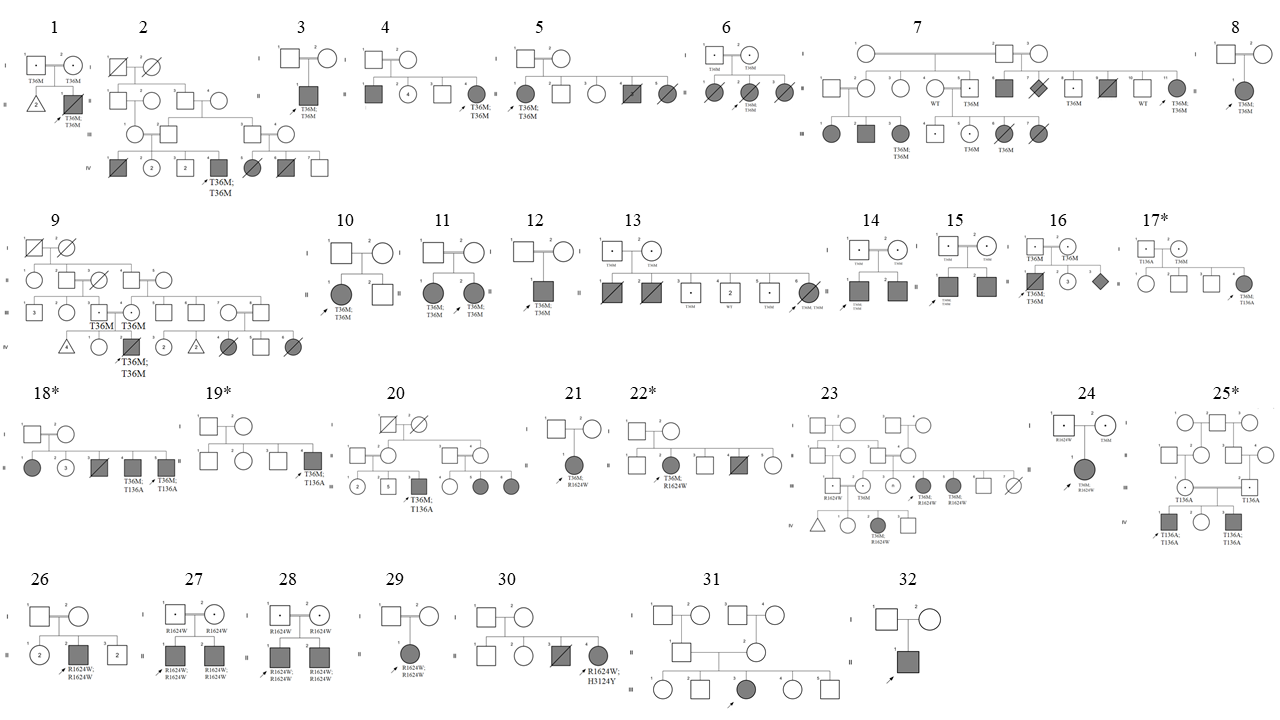
**

Figure S1: Pedigrees of the 32 analysed families.

Squares represent males, circles represent females. Filled symbols indicate the affected status. Double-horizontal bars illustrate parental consanguinity. F: Family. Genetically solved families are F1-F30. Unsolved families are F31-F32. *Patients had been previously described in [12], where 17 is P25, 18 is P27, 19 is P44, 22 is P53 and 25 is P22.


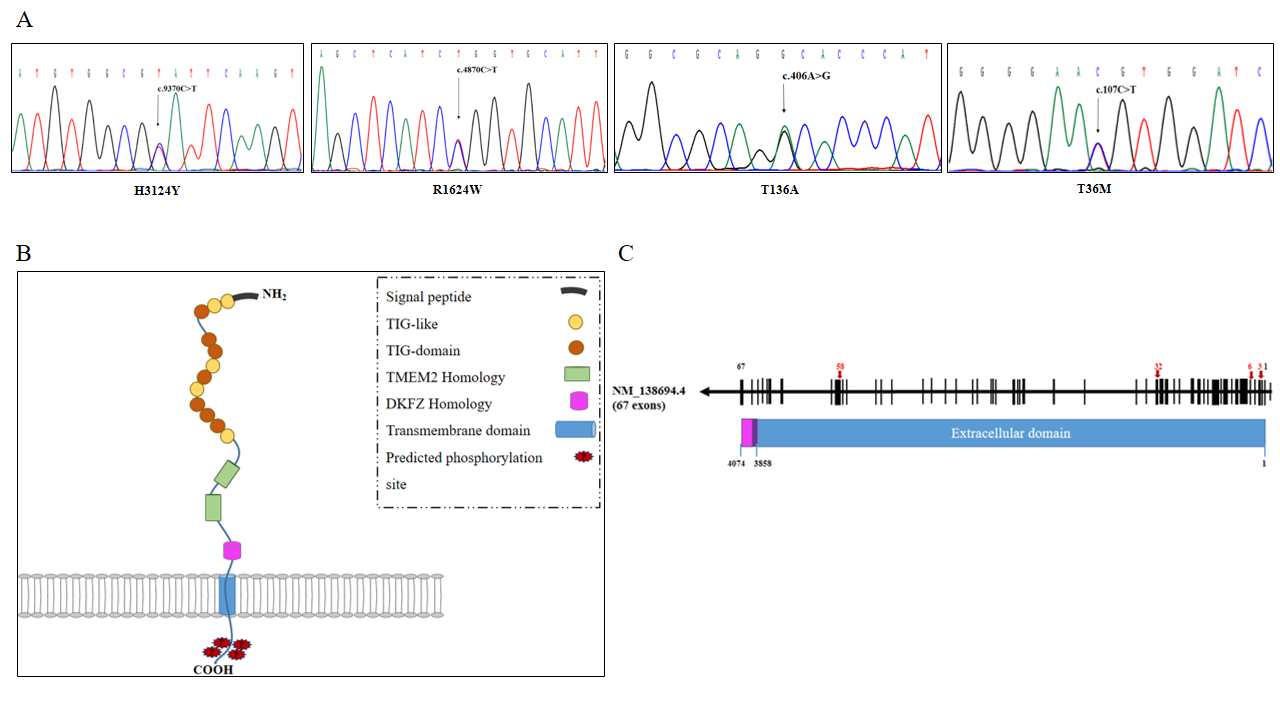


Figure S2. Representation of the missense variants of the *PKHD1* gene detected in ARPKD patients in relation to the gene exon structure and protein domains.

A. Chromatograms of the identified mutations in the *PKHD1* gene. Heterozygous sequence variants are arrowed with the nucleotide changes indicated (reference sequence NM_138694.4)

B. Predicted protein domains of fibrocystin, with its large extracellular domain, single transmembrane domain, and short cystoplasmic tail.

C. Simplified structure of the *PKHD1* transcript (longest open reading frame) (NM_138694.4) consisting of 67 exons that encode for a 4074 amino acid. Mutations are identified by red arrows.

**Supplementary Tables**

Table S1. Disease categories and genes selected for targeted NGS panel for cystic kidney disease.

| **Diseases** | **Gene** |
| --- | --- |
| Autosomal dominant polycystic kidney disease (ADPKD) | *PKD1, PKD2* |
| Autosomal recessive polycystic kidney disease (ARPKD) | *PKHD1* |
| Autosomal dominant tubulo interstitial kidney disease (ADTKD) | *HNF1B, REN, UMOD* |
| Nephronophthisis (NPHP) | *NPHP1, INVS, NPHP3, NPHP4, GLIS2, NEK8, TMEM67, TTC21B, WDR19, ZNF423, CEP164, ANKS6, CEP83, DCDC2, SDCCAG8 & CEP290* |
| Medullary cystic kidney disease | *UMOD, MUC1* |
| Meckel-Gruber syndrome (MKS) | *MKS1, TMEM67, CEP260, NPHP3 & RPGRIP1L* |
| Bardet-Biedl syndrome (BBS) | *BBS1, BBS2, ARL6, BBS4, BBS5, MKKS, BBS7, TTC8, BBS9, BBS10, TRIM32, BBS12, MKS1, CEP290, WDPCP, SDCCAG8, LZTFL1, BBIP1, IFT27* |
| Autosomal dominant Polycystic liver disease (ADPLD) | *PRKCSH, SEC63* |
| Joubert syndrome (JBTS) | *AHI1, NPHP1, CEP290, TMEM67, RPGRIP1L & TTC21B* |
| Renal cysts and diabetes syndrome | *HNF1B* |
| Renal dysplasia, cystic susceptibility | *BICC1* |

Table S2. Primers used for PCR amplification and sequencing of *PKHD1* gene.

| **Primer Name** | **Gene - Exon** | **Sequence 5'--> 3'** |
| --- | --- | --- |
| PKHD1_3F | *PKHD1* Exon 3 | CTGAGGCAGGTTAAATATTGCTT |
| PKHD1_3R | *PKHD1* Exon 3 | GTCTGTTCGTCTCCCTTCAGG |
| PKHD1_6F | *PKHD1* Exon 6 | GTGCCTCCTGTGTTTGTGAA |
| PKHD1_6R | *PKHD1* Exon 6 | TCCAGTCTCCAACATCAACTCA |
| PKHD1_32AF | *PKHD1* Exon 32 | AACACATGCCCTACCTTCCA |
| PKHD1_32AR | *PKHD1* Exon 32 | AACATCACAGTTCAGGTTCCC |
| PKHD1_32BF | *PKHD1* Exon 32 | GAAGTAACCTCTCCAACTCAGTC |
| PKHD1_32BR | *PKHD1* Exon 32 | CCACAAATACCATCGGCTCAT |
| PKHD1_32CF | *PKHD1* Exon 32 | TCTCTGACCACTGTGCTGAT |
| PKHD1_32CR | *PKHD1* Exon 32 | TGAAACACTTGGGGCATAATGT |
| PKHD1_32DF | *PKHD1* Exon 32 | TGATTAGGGGTCAGAGGTTAGC |
| PKHD1_32DR | *PKHD1* Exon 32 | CCACTGCAAAGGTTAAGATGTCA |
| PKHD1_32EF | *PKHD1* Exon 32 | AGGTAGATGGACTTTGGTATCACA |
| PKHD1_32ER | *PKHD1* Exon 32 | TTTCCAGAAGTGAAAGGAGCTAC |
| PKHD1_58AF | *PKHD1* Exon 58 | TCAGCCTTTTGTGGGGAAGA |
| PKHD1_58AR | *PKHD1* Exon 58 | TGAAAGCCAAGAAGCCAGAG |
| PKHD1_58BF | *PKHD1* Exon 58 | GCCTTCATCTCTATAAGGAAAGTGG |
| PKHD1_58BR | *PKHD1* Exon 58 | TGCATGGATGTATGAAATGGCA |

Table S3. Different *PKHD1* founder mutations associated with different ethnicities.

| Origin | Nucleotide change | aa change | Exon | Mutation Type | References |
| --- | --- | --- | --- | --- | --- |
| Finnish | c.1486C>T | p.R496* | 16 | Nonsense | Bergmann et al., 2003 |
|  | c.10412T>G | p.V3471G | 61 | Missense |  |
| French | c.7350+653A>G | p.G2451fs*18 | IVS46 | Intronic/Pseudo exon activation | Michel-Calemard et al., 2009 |
| Ashkenazi | c.3761_3762delCCinsG | p.A1254Gfs*49 | 32 | Frameshift/Indel or Duplication | Quint et al., 2016 |
| Afrikaner | c.1880 T>A | p.M627K | 20 | Missense | Lambie et al., 2015 |
