# Supplementary figures and images for: Clinical and genetic characteristics of autosomal recessive polycystic kidney disease in Oman
Source: BMC Nephrol. 2020 Aug 14;21:347. doi: 10.1186/s12882-020-02013-2 (PMC7429752; doi:10.1186/s12882-020-02013-2)

## Slide 1
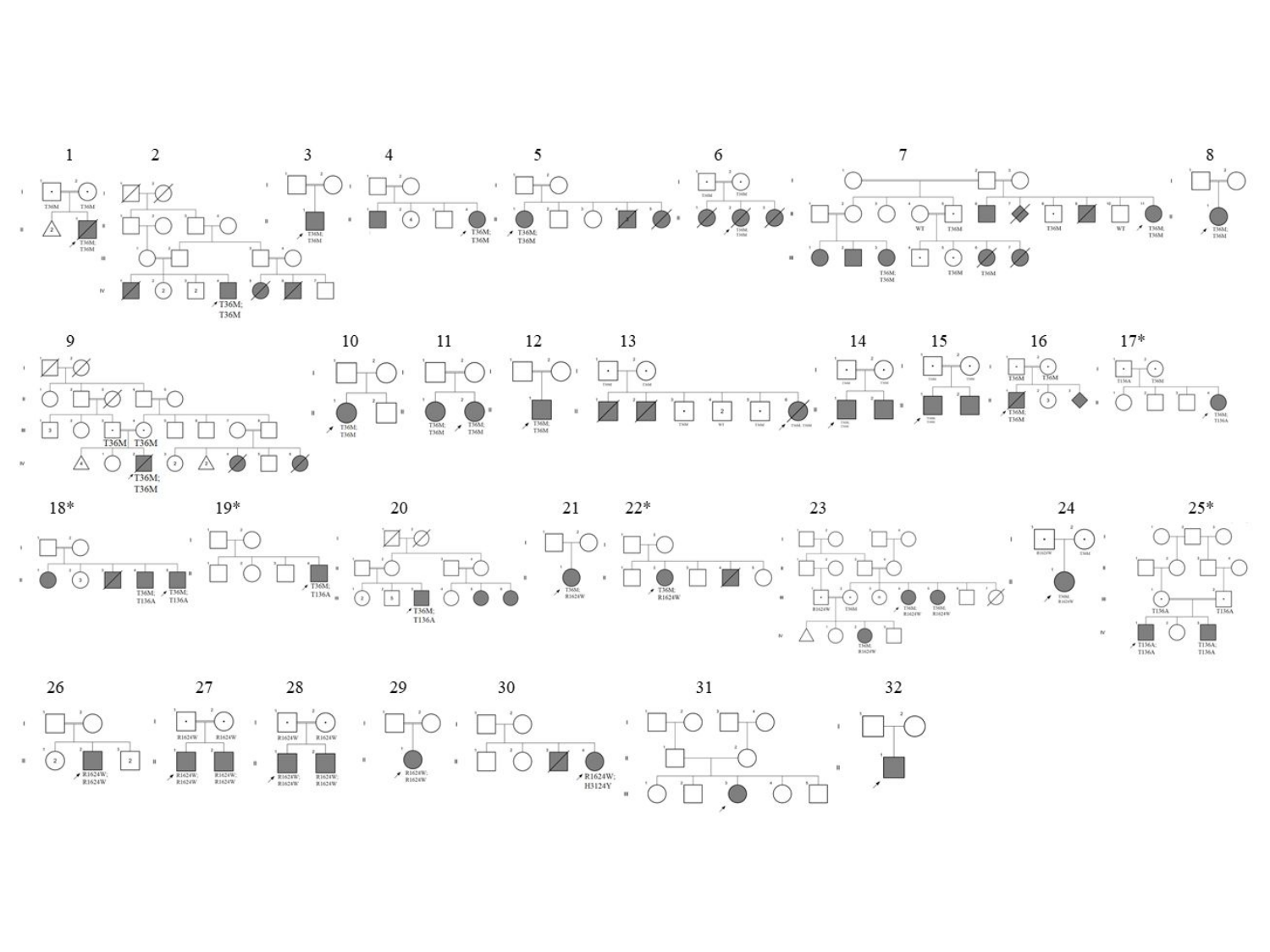

Supplement: Supplementary file 1 — Additional file 1 : Figure S1. Pedigrees of the 32 analysed families. Figure S2. Representation of the missense variants of the PKHD1 gene detected in ARPKD patients in relation to the gene exon structure and protein domains. Table S1. Disease categories and genes selected for targeted NGS panel for cystic kidney disease. Table S2. Primers used for PCR amplification and sequencing of PKHD1 gene. Table S3. Different PKHD1 founder mutations associated with different ethnicities. [file 12882_2020_2013_MOESM1_ESM.zip › Figure S1R2.pptx]

## Slide 1
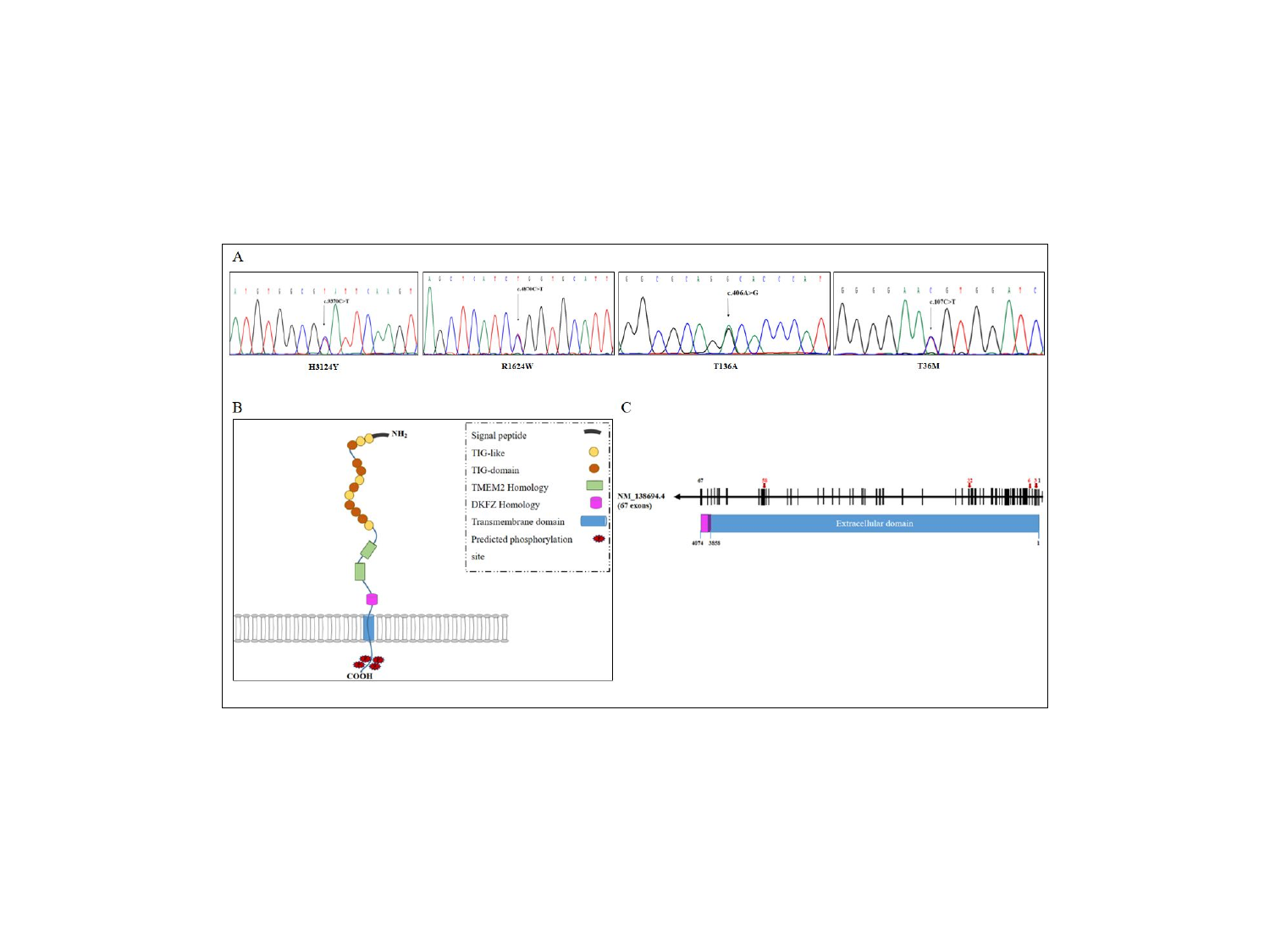

Supplement: Supplementary file 1 — Additional file 1 : Figure S1. Pedigrees of the 32 analysed families. Figure S2. Representation of the missense variants of the PKHD1 gene detected in ARPKD patients in relation to the gene exon structure and protein domains. Table S1. Disease categories and genes selected for targeted NGS panel for cystic kidney disease. Table S2. Primers used for PCR amplification and sequencing of PKHD1 gene. Table S3. Different PKHD1 founder mutations associated with different ethnicities. [file 12882_2020_2013_MOESM1_ESM.zip › Figure S2R2.pptx]
